# Oral Efficacy of a Diselenide Compound Loaded in Nanostructured Lipid Carriers in a Murine Model of Visceral Leishmaniasis

**DOI:** 10.1021/acsinfecdis.1c00394

**Published:** 2021-11-12

**Authors:** Mikel Etxebeste-Mitxeltorena, Esther Moreno, Manuela Carvalheiro, Alba Calvo, Iñigo Navarro-Blasco, Elena González-Peñas, José I. Álvarez-Galindo, Daniel Plano, Juan M. Irache, Antonio J. Almeida, Carmen Sanmartín, Socorro Espuelas

**Affiliations:** † Institute of Tropical Health, Department of Pharmaceutical Technology and Chemistry, School of Pharmacy and Nutrition, University of Navarra, 31008 Pamplona, Spain; ‡ Instituto de Investigación Sanitaria de Navarra (IdiSNA), 31008 Pamplona, Spain; § Research Institute for Medicines (iMed.ULisboa), Faculty of Pharmacy, Universidade de Lisboa, 1649-003 Lisbon, Portugal; ∥ Department of Chemistry, School of Sciences, University of Navarra, 31008 Pamplona, Spain; ⊥ Department of Pharmaceutical Technology and Chemistry, School of Pharmacy and Nutrition, University of Navarra, 31008 Pamplona, Spain; ∇ Department of Pharmaceutical Technology and Chemistry, School of Pharmacy and Nutrition, University of Navarra, 31008 Pamplona, Spain

**Keywords:** diselenide, nanostructured lipid carriers, visceral leishmanisis, *L. infantum*, oral treatment

## Abstract

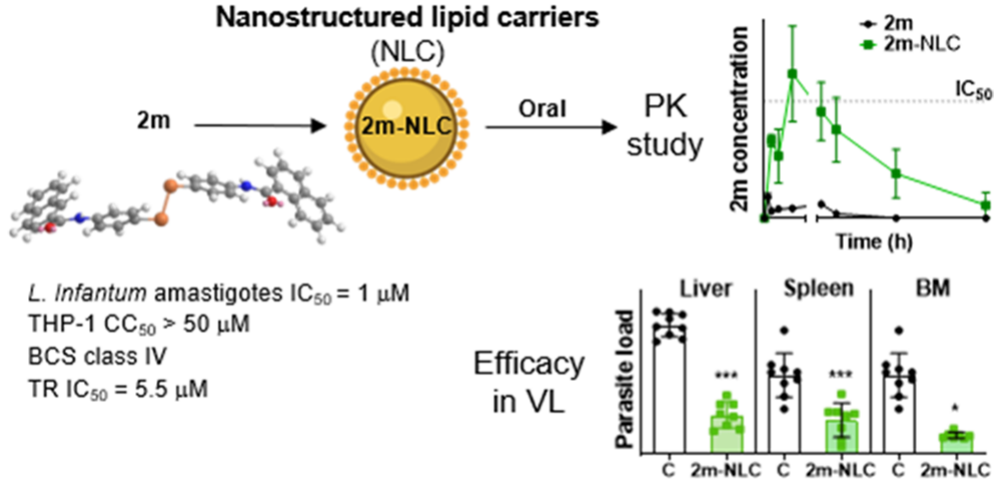

Leishmaniasis urgently needs new oral treatments, as it is one of the most important neglected tropical diseases that affects people with poor resources. The drug discovery pipeline for oral administration currently discards entities with poor aqueous solubility and permeability (class IV compounds in the Biopharmaceutical Classification System, BCS) such as the diselenide **2m**, a trypanothione reductase (TR) inhibitor. This work was assisted by glyceryl palmitostearate and diethylene glycol monoethyl ether-based nanostructured lipid carriers (NLC) to render **2m** bioavailable and effective after its oral administration. The loading of **2m** in NLC drastically enhanced its intestinal permeability and provided plasmatic levels higher than its effective concentration (IC_50_). In *L. infantum-*infected BALB/c mice, **2m**-NLC reduced the parasite burden in the spleen, liver, and bone marrow by at least 95% after 5 doses, demonstrating similar efficacy as intravenous Fungizone. Overall, compound **2m** and its formulation merit further investigation as an oral treatment for visceral leishmaniasis.

Leishmaniasis comprises a group of parasitic diseases caused by protozoa of the genus *Leishmania*.^1^ Among the different clinical manifestations, visceral leishmaniasis (VL) is the most severe form of the disease, and it is principally caused by the species *L. infantum* and *L. donovani*. This parasite resides in host macrophages, mainly from liver, spleen, bone marrow, and lymph nodes, and it causes anemia, leukopenia, hepatosplenomegaly, hypoalbuminemia, weight loss, and ultimately death if untreated.^1^ 300,000 out of more than 1 million reported cases of leishmaniasis every year belong to VL, which is responsible for around 40,000 deaths.^2^

The range of drugs available for the treatment of VL is limited, and it includes pentavalent antimonials, amphotericin B deoxycholate, lipid formulations of amphotericin B, miltefosine, and paromomycin.^3,4^ All of them have limitations in terms of toxicity, variable efficacy, price, and inconvenient treatment schedules.^5^ Moreover, they are parenteral drugs, with the exception of miltefosine, which is orally administered. Nevertheless, miltefosine includes several limitations such as gastrointestinal toxicity, teratogenicity, high cost, and long elimination half-life, leading to subtherapeutic levels over several weeks and facilitating the appearance of resistances.^6^ Due to the geographical distribution of the disease and the poor access to drugs for those people infected in low-middle income countries, it is a priority the search of new drugs and/or formulation strategies to be orally administered.^7^

Although the combination therapy of drugs has improved the VL outcome of current therapeutic arsenal, the long-term goal is to identify new active compounds and develop an entirely new generation of oral drugs.^8^ Drug discovery algorithms for oral administration currently recommend discarding entities with poor aqueous solubility and/or permeability because they lead to low bioavailability and suboptimal efficacy. However, lipid-based systems have shown marked increase in the oral absorption of this type of compound.^9,10^ Among the different kinds of lipid systems, nanostructured lipid carriers (NLC) stand out over other types of nanocarriers because they offer many encouraging advantages.^11^ Compared with liposomes or nanoemulsions, NLC present higher stability in the physiological conditions found in the gastrointestinal tract.^12^ Moreover, they overcome the inherent disadvantages of their parent solid lipid nanoparticles (SLN), such as low drug payload and drug expulsion during storage.^13^

In the past years, our group has reported the synthesis and *in vitro* antileishmanial activity of several series of selenocompounds.^14−19^ Two of these derivatives, *N,N′*-(4,4′-diselanediylbis(4,1-phenylene))bis-furan-2-carboxamide (**2h**) and *N,N’*-(4,4′-diselanediylbis(4,1-phenylene))bisnaphthamide (**2m**) (Figure 1), could be considered a great starting point in order to profile good hit compounds for leishmaniasis treatment, as they presented IC_50_ values lower than 3 μM against *L. infantum* amastigotes and selectivity indexes around 23 and 50, respectively (Table 1).^19^ Their mechanism of action has not yet been properly determined, although the increase in the intracellular thiol levels and the inhibition of the trypanothione reductase (TR) activity could implicate alterations in the parasite redox metabolism. TR is a validated target for the designing of antileishmanial drugs.^20^ It is essential for parasite survival, but it is absent in the host, in which TR is replaced by glutathione reductase.^21^ Moreover, antimonials currently in clinical use inhibit TR and evidence that TR is a druggable target. Although a large number of TR inhibitors have been identified by virtual and high-throughput screening,^22,23^ few of them have further progressed because of the need for strong and irreversible inhibition in the presence of endogenous substrates and suitable drug-like properties.^24^

**Figure 1:**
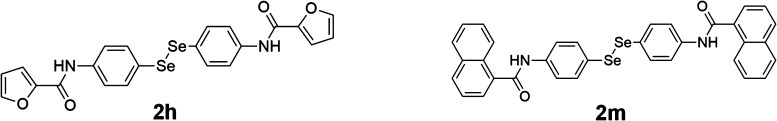
Structures of compounds **2h** and **2m** selected for further studies.

**Table 1:** Activity of **2h** and **2m** Compounds against *L. infantum* Amastigotes and TR, and Cytotoxic Activity on THP-1 Cells^19^

compound	intramacrophage *L. infantum*amastigotes IC_50_^b^ (μM)	THP-1 cells CC_50_^c^ (μM)	selectivity index	TR^a^ inhibition IC_50_^b^ (μM)
**2h**	2.2 ± 0.8	>50	>20	14.0 ± 0.9
**2m**	1.0 ± 0.2	>50	>50	5.5 ± 0.1

a Trypanothione reductase.

b Concentration that inhibits parasite growth or TR activity by 50%.

c Concentration that kills cells by 50%.

In this study, compounds **2h** and **2m** were put through the screening cascade for VL therapy.^25,26^ The *in vitro* absorption, distribution, metabolism, and excretion (ADME) properties to predict their oral bioavailability, namely, intestinal permeability, metabolic stability, and *in vivo* pharmacokinetics, were determined. Besides, to circumvent **2h** and **2m** poor water solubility and to enhance their oral permeability and bioavailability, their encapsulation in NLC was carried out. Finally, their efficacy was assayed in a murine model of established VL when administered orally.

## Results

We recently published the synthesis and *in vitro* antileishmanial activity of novel selenocompounds whose mechanism of action mainly involved parasite redox processes.^19^ Compounds **2h** and **2m** were chosen for further profiling because they exhibited the highest selectivity index, calculated as the ratio between the activity against intramacrophage parasites and the cytotoxicity against host macrophages (THP-1 cells) (Table 1), among the different synthesized compounds. Furthermore, both compounds fulfilled hit criteria in drug discovery for VL, as they showed an *in vitro* IC_50_ < 10 μM and good selectivity index.^25,26^

### *In Vitro* ADME Properties for Compounds **2h** and **2m**: Acceptable Metabolic Stability but Unsuitable for Oral Bioavailability

Prior to *in vivo* studies, the *in vitro* ADME properties of **2h** and **2m** likely to be predictive of their oral bioavailability were measured. Both compounds exhibited low aqueous solubility in fasted simulated intestinal fluid (SSIF) and very poor *ex vivo* intestinal permeability (Table 2). However, **2h** and **2m** showed acceptable metabolic stability in mouse liver microsomes, as indicated by their metabolism half-time (*t*_1/2_) and intrinsic clearance (Cl_int_) values of 1.7 h and 24.9 mL·min^–1^·kg^–1^ for **2h** and 1.2 h and 33.7 mL·min^–1^·kg^–1^ for **2m**, respectively (Table 2 and Figure S1). In general, compounds with *t*_1/2_ > 1 h are defined as stable in liver microsomal metabolism, and Cl_int_ values between 15 and 45 mL·min^–1^·kg^–1^ are indicators of intermediate clearance rate.^27^

**Table 2:** *In Vitro* ADME Properties Determined for Compounds **2h** and **2m**^a^

compound	microsomal stability*t*_1/2_^b^ (h)	Cl_int_^c^ (mL/min·kg)	solubility in SSIF^d^ (μg/mL)	intestinal permeability *P*_app_^e^ (×10^–7^) cm/s
**2h**	1.7	24.9	3.2 ± 1.7	7.2 ± 2.6
**2m**	1.2	33.7	2.5 ± 0.5	5.9 ± 1.1

a Values are the means ± SD of at least three independent measurements.

b Half-time.

c Intrinsic clearance.

d Fasted simulated intestinal fluid.

e Apparent permeability.

### Loading of Compounds **2h** and **2m** into NLC: Preparation and Characterization

**2h** (log*P* value of 1^19^) and especially **2m** (log*P* 5^19^) are poorly water-soluble compounds and, thus, *a priori* suitable candidates for their loading into NLC.^28^ Moreover, these types of nanocarriers have widely demonstrated potential to increase solubility, oral bioavailability, and even lymphatic absorption of hydrophobic drugs.^11^ The poor solubility of **2h** and **2m** compounds in many common organic solvents and lipids restricted NLC fabrication and composition to the hot high-shear homogenization (HHSH) process with glyceryl palmitoestearate (Precirol) and diethylene glycol monoethyl ether (Transcutol) as solid and liquid lipids, respectively. Transcutol was the solvent that presented the highest solubilizing capability for **2h** and **2m** compounds, being the only one able to dissolve up to 6 mg of compound in 0.4 g. The composition of the optimized NLC is shown in Table 3.

**Table 3:** Composition of Aqueous NLC Dispersions Containing Compounds **2h** and **2m**

		Composition (% w/v)	
		**2h**-NLC	**2m**-NLC
Solid lipid	Precirol ATO5	12	12
Liquid lipid	Transcutol HP	5	5
Surfactant	Tween 80	20	20
Compound	**2h**	0.093	-
Compound	**2m**	-	0.089
Water		63	63

Homogeneous NLC (polydispersity index, PDI < 0.2) with a mean size around 100 nm and high encapsulation efficiencies (between 85% and 95%) were obtained (Table 4) for both compounds. However, the payload (around 6 mg compound/g lipids, Table 4) could be considered as low. Negative zeta potential values of −14 mV and −16 mV were observed for **2h**-NLC and **2m-**NLC, respectively. Although they are below the critical zeta potential for stability in terms of purely electrostatic repulsions, the sterically stabilizing effect of polysorbate 80 (Tween 80) could explain the low PDI of the NLC suspensions. In fact, a relatively high concentration of Tween 80 was required to obtain monodisperse NLC of around 100 nm by the HHSH method with a total lipid concentration of 17%. In general, NLC sizes and PDI tended to increase with lipid concentration.^29,30^

**Table 4:** Physicochemical Properties of Unloaded Aqueous NLC and **2h** and **2m** Loaded NLC Dispersions (**2h**-NLC and **2m**-NLC)^a^

formulation	size (nm)	PDI^b^	zeta potential (mV)	EE^c^ (%)	DL^d^ (μg/mg lipids)
NLC	120 ± 12	0.2	–17 ± 1	-	-
**2h**-NLC	110 ± 21	0.2	–14 ± 1	93 ± 4	0.56 ± 0.02
**2m**-NLC	125 ± 33	0.2	–16 ± 1	89 ± 4	0.54 ± 0.02

a Values are the means ± SD of at least three independent measurements.

b Polydispersity index.

c Encapsulation efficiency.

d Drug loading.

**Figure 2:**
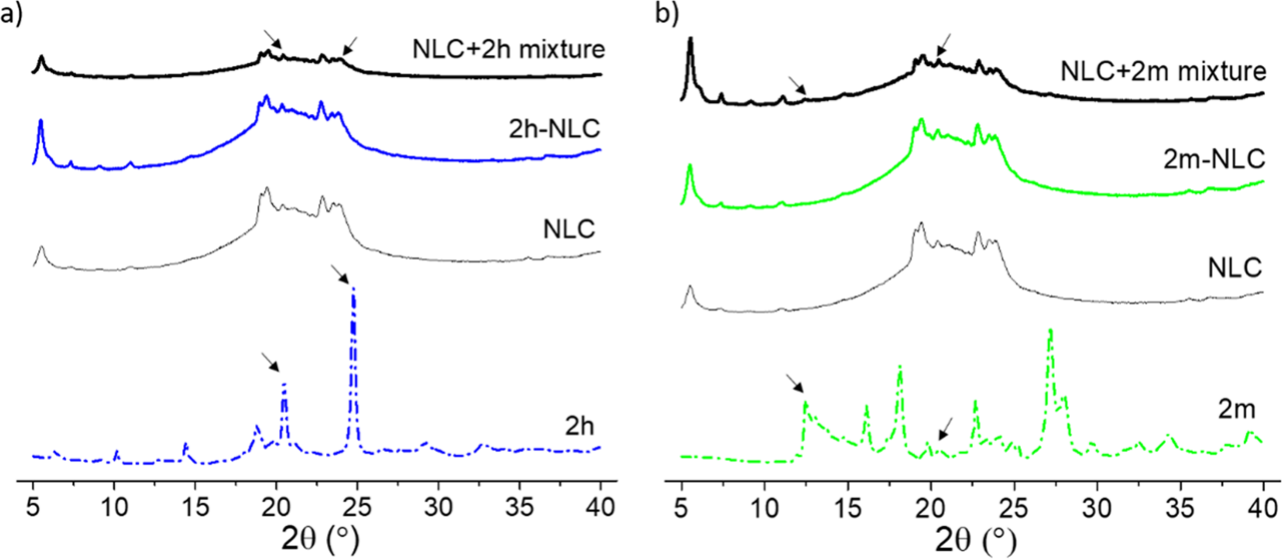
X-ray diffraction patterns of (a) pure **2h**, empty NLC, **2h**-NLC, and the physical mixture of **2h** and empty NLC, and (b) pure **2m**, empty NLC, **2m**-NLC, and the physical mixture of **2m** and empty NLC. Physical mixtures were prepared in equivalent concentration as in the final optimized formulations.

**Figure 3:**
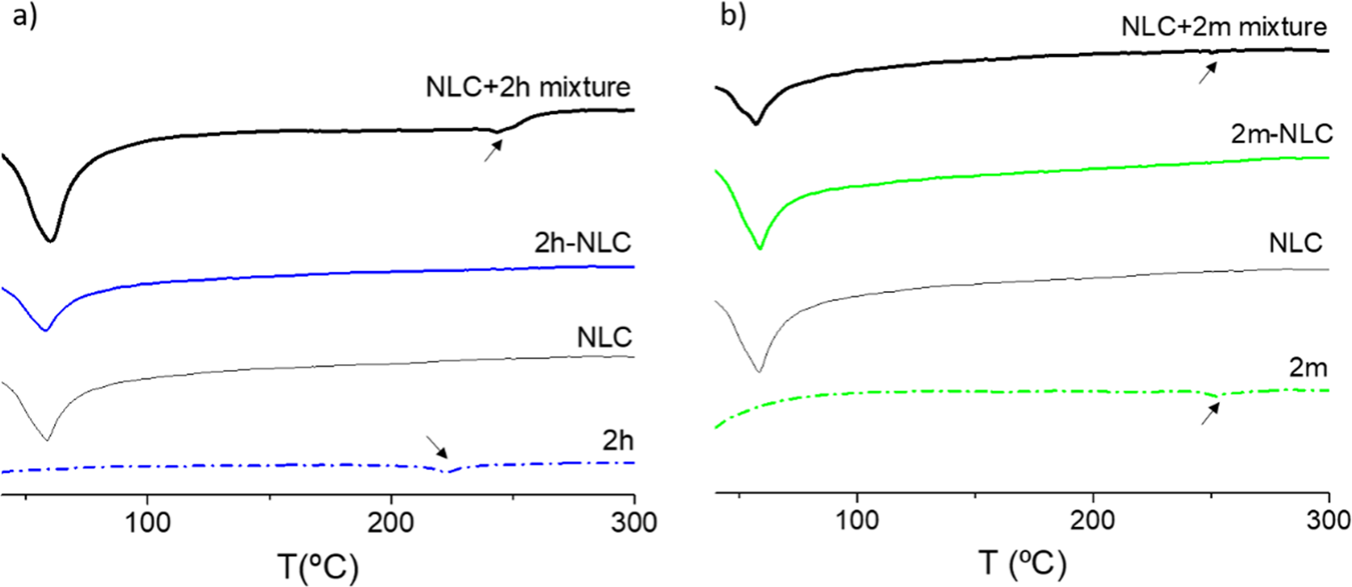
Thermal curves of (a) pure **2h**, empty NLC, **2h**-NLC, and the physical mixture of **2h** and empty NLC; and (b) pure **2m**, empty NLC, **2m**-NLC, and the physical mixture of **2m** and empty NLC. Physical mixtures were prepared in equivalent concentrations as in the final optimized formulations.

TG-DTA and X-ray diffraction studies were performed to analyze the degree of crystallinity, the possible polymorphic modifications, and the drug incorporation into the lipid matrix, as these parameters could modify their release profile and affect the stability of the system over time.^29,30^ Thermal curves and X-ray diffraction patterns of pure **2h**, pure **2m**, empty NLC, **2h**-NLC, **2m**-NLC, and their physical mixtures in equivalent concentration (0.3% w/w) as in the final optimized formulations are shown in Figure 2 and Figure 3. Thermal curves of pure **2h** and **2m** compounds showed melting endothermic peaks at approximately 222 and 252 °C, respectively. Empty NLC showed a sharp melting peak at 60 °C corresponding to Precirol. **2h**-NLC (Figure 2a) and **2m**-NLC (Figure 2b) formulations presented a similar profile to that of empty NLC. They did not show the characteristic melting peaks of the pure compounds. However, these endothermic peaks could be detected in their physical mixture with empty NLC, although with an increase in the case of compound **2h** (from 222 °C for **2h** to 245 °C in **2h** + NLC physical blend).

Regarding X-ray diffraction studies, compound **2h** exhibited two sharp peaks at 2θ of 20.5° and 24.8°, respectively. These peaks were still discernible in its physical mixture with empty NLC, whereas they were not present in the **2h**-NLC formulation (Figure 3a). Besides, **2m** displayed a crystalline structure with multiple peaks in its X-ray diffraction pattern. However, although these peaks were not found in the **2m**-NLC formulation, peaks at 2θ of 12.5° and 20.5°, respectively, were still detected in the **2m** and NLC physical mixture (Figure 3b).

Overall, the absence of peaks corresponding to the melting transitions of pure compounds or XRD patterns in **2m**-NLC and **2m**-NLC formulations (whereas some signals could be still visible in the corresponding physical mixtures) probably reflect the incorporation of the compounds into an amorphous or molecularly dispersed state inside the lipid matrix.

In order to prolong their storage, aqueous dispersions of NLC were lyophilized. Trehalose (at a ratio 1:1.5 to lipid) was suitable for protecting NLC of aggregation, as lyophilized NLC with this cryoprotector showed similar size, drug loading, and acceptable PDI (between 0.2 and 0.3).

### Slow Release Profile of Compounds **2h** and **2m** from NLC

**Figure 4:**
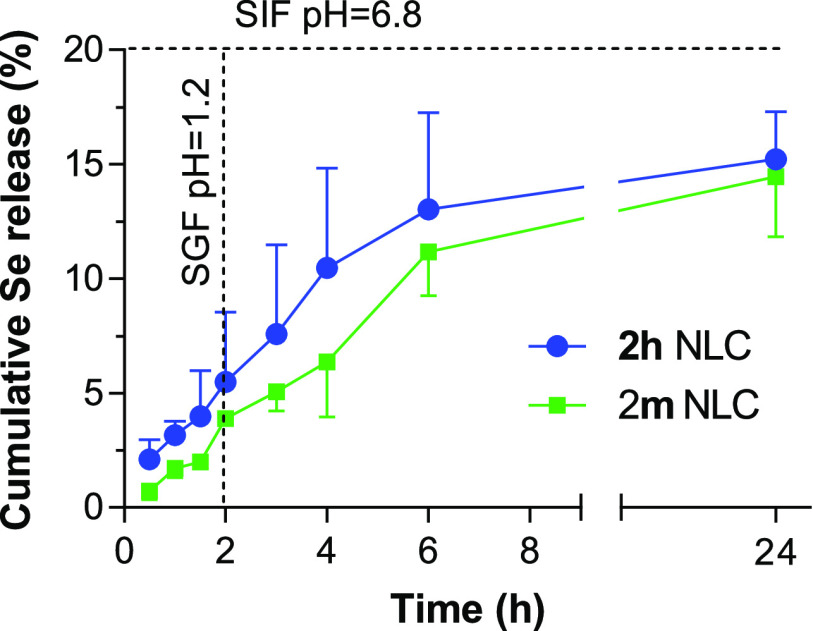
**2m** and **2m** release profiles from NLC as a function of time after incubation in SGF (0–2 h) and SIF (2–24 h) at 37 °C under sink conditions and expressed as percentage of cumulative Se release. Data are expressed as the mean ± SD, *n* = 3.

Figure 4 represents the release profile of **2h** and **2m** compounds from NLC as a function of time when incubated in simulated gastric fluid (SGF, during 2 h) and later in simulated intestinal fluid (SIF, during at least 4 h further) in order to mimic the oral administration. Both formulations exhibited a slow and incomplete release profile. **2h** was released from NLC slightly faster than **2m**, although the differences were not significant. About 5% of the compounds (5.5 ± 3.1% for **2h** and 3.9 ± 0.3 for **2m**) was released in the first 2 h of incubation in SGF (mean residence time in the stomach). Then, the slow release continued in SIF. After 4 h of incubation in SIF, NLC released around 13% and 10% of **2h** and **2m**, respectively. The rapid degradation of several types of Precirol-based NLC has been previously described,^31^ although the correlation between degradation and drug release has not been properly evaluated. We could hypothesize that the degradation of NLC into mixed micelles solubilizing the drug and unable to cross the dialysis membrane may be responsible for the low amount of compound (around 15%), expressed as selenium (Se) content, released at the end of the study (24 h).

### Loading of compounds **2h** and **2m** into NLC Enhances Their Oral Permeability and Oral Biovailability

**Figure 5:**
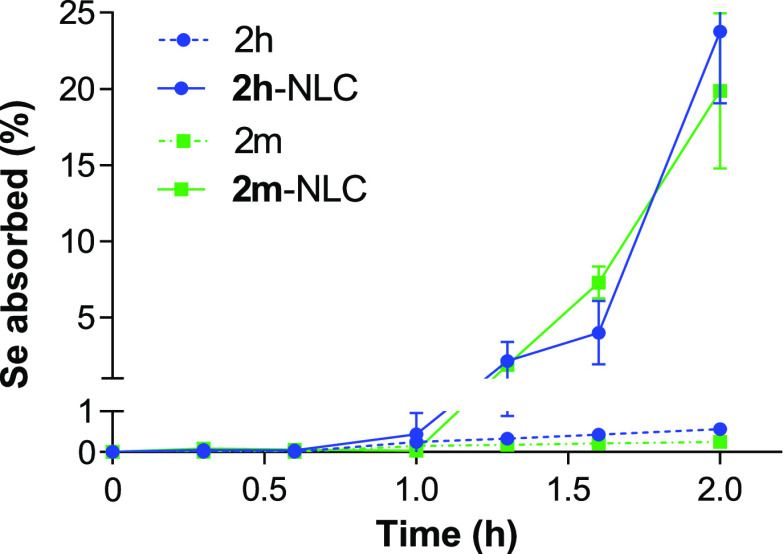
Percentage of the dose of free compounds (**2h** and **2m**) and compounds formulated in NLC (**2h**-NLC and **2m**-NLC), measured as Se content, absorbed across rat jejunum segments mounted in Ussing chambers plotted against time for mucosal-to-serosal transport (absorptive direction). Data are expressed as mean ± SD, *n* = 4.

Figure 5 represents the percentage of the dose of Se absorbed through jejunum portions in the Ussing chambers plotted against time. Either free or encapsulated into NLC, the absorption of compounds through the intestine was characterized by an average lag time of 1 h. However, the loading of **2h** and **2m** into NLC produced a higher increase in the intestinal permeability compared to free drugs. At the end of the study, the amount of Se in the receptor compartment (RC) for the nanoparticle formulations was 20 times higher than for the free compounds, with apparent permeability (*P*_app_) values of (7.67 ± 1.85) × 10^–5^ and (6.44 ± 1.08) × 10^–5^ cm/s for **2h-**NLC and **2m-**NLC, respectively. Estimated *P*_app_ values for the free compounds were 100-fold lower, around (7.17 ± 2.62) × 10^–7^ and (5.91 ± 1.08) × 10^–7^ cm/s for **2h** and **2m**, respectively (Table 2). It is generally accepted that Caco-2 cell permeability coefficients higher than >10^–6^ cm/s represent high permeability.^32^

**Figure 6:**
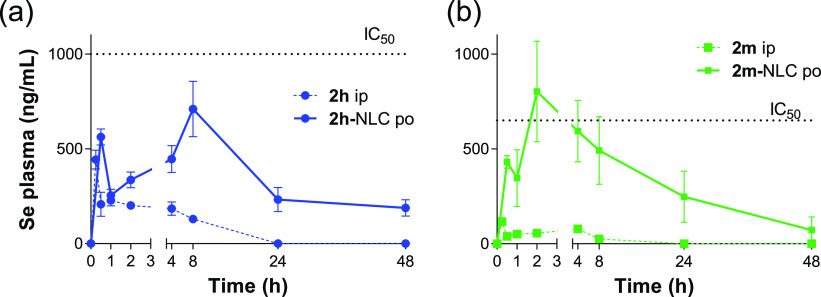
Plasma concentration–time curve of Se in BALB/c mice after a single i.p. or p.o. administration of compounds at 4 mg/kg either in their free form (**2h** and **2m**, i.p.) or loaded into NLC (**2h**-NLC and **2m**-NLC. p.o.). Data represents mean ± SD, *n* = 3.

A single i.p. or p.o. dose of compounds **2h** and **2m**, either free or encapsulated in NLC, was administered to mice to estimate the oral bioavailability and, thus, the potential of these compounds as p.o. candidates for the treatment of VL. The dose of 4 mg/kg was selected after determining a LD_50_ value of 17.5 mg/kg for **2h** and **2m**, respectively, by the i.p. route. The main pharmacokinetic (PK) parameters, calculated by noncompartmental analysis, are summarized in Table 5. No Se above basal level was quantified after the p.o. administration of free selenocompounds. However, after the oral administration of NLC, Se plasma concentration was significantly higher than the values obtained after the i.p. administration of the solubilized free compounds. This increase was accompanied by a delay of the time of maximum concentration (*t*_max_) and higher values of elimination half-times (*t*_1/2_). Apparent clearance (Cl) values, after the p.o. administration, were referred to total administered dose because mice immediately died after i.v. bolus of the compounds. These Cl values allowed the determination of hepatic clearance rates (<0.3) suitable for further biological evaluation, except for i.p. **2m** compound that presented an extraction ratio (ER) higher than 0.7 (Table 5). However, we must be very cautious with these PK parameters, because they are probably estimated from parent compounds and metabolites, as Se plasma levels and not the compounds were measured. Metabolism probably also explains the lack of correlation between predicted hepatic clearance from *in vitro* liver microsomal stability studies and *in vivo* data. In fact, both compounds either free (i.p.) or loaded into NLC (p.o.) displayed several peaks in the plasma Se concentration–time curve (Figure 6). After oral administration of NLC, the first peak concentration of Se in plasma was reached at 0.5 h. After the initial absorption phase, the mean plasma concentration of Se displayed the highest peak at 8 h for **2h**-NLC and 2 h for **2m**-NLC, followed by a slower decline. In any case, only following oral administration of **2m**-NLC did the Se plasma concentration reach values above its *in vitro* intramacrophage amastigote activity (IC_50_ of 1 μM) (Table 1).

**Table 5:** Pharmacokinetic Parameters of Compounds in BALB/c Mice after a Single i.p. (**2h** and **2m**) or p.o. (**2h**-NLC and **2m**-NLC) Administration at 4 mg/kg^a^

parameter	**2h** i.p.	**2h**-NLC p.o.	**2m** i.p.	**2m-**NLC p.o.
AUC_0-t_^b^ (ng·h/mL)	1935.7 ± 737.2	14,915.0 ± 4325.8**	353.7 ± 179.6	14,382.7 ± 2806.2***
*C*_max_^c^ (ng/mL)	443.0 ± 50.6	649.7 ± 146.7	115.3 ± 15.3	700.7 ± 262.2**
*t*_max_^d^ (h)	0.3 ± 0.0	8.0 ± 0.0****	0.3 ± 0.0	2.2 ± 1.8
*t*_1/2_^e^ (h)	7.9 ± 2.9	29.1 ± 7.8*	3.2 ± 0.2	20.7 ± 11.9
Cl^f^ (mL/min/kg)	24.5 ± 10.9	3.1 ± 0.7	123.7 ± 42.8	4.1 ± 0.9***
MRT_0-*t*_^g^ (h)	10.9 ± 3.9	43.6 ± 10.2*	5.1 ± 0.3	23.6 ± 17.1
ER^h^	<0.3	<0.3	>0.7	<0.3

a Data expressed as mean ± SD, *n* = 3. *p < 0.05. **p < 0.001. ***p < 0.001. ****p < 0.0005, using a nonparametric U-Mann–Whitney test.

b Area under the concentration–time curve.

c Maximum plasma concentration.

d Time of maximum concentration.

e Half-life.

f Clearance rate. Cl was apparent clearance estimated from the administered dose.

g Mean residence time.

h Extraction ratio. Results analyzed by unpaired *p* < 0.01 *t* test between free compounds and compounds loaded into NLC.

### p.o. Administration of **2m**-NLC Showed Similar Efficacy as i.v. Fungizone in *L. infantum*-Infected Mice

**Figure 7:**
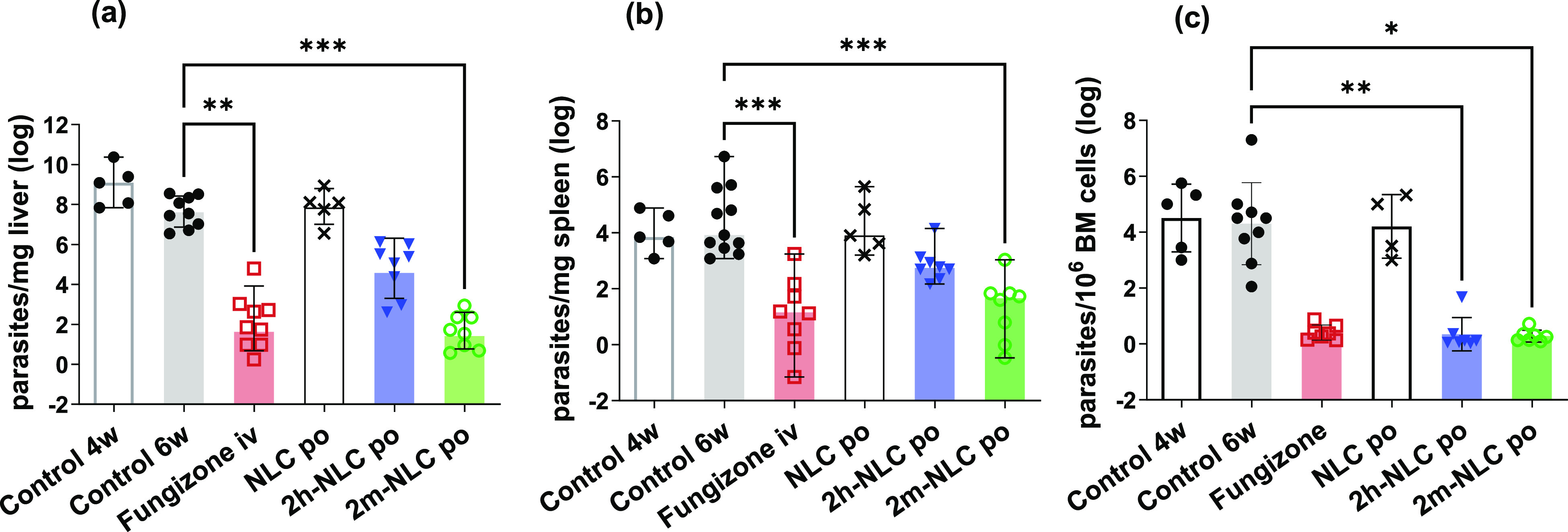
Parasite burden in the liver (a), spleen (b), and bone marrow (BM, c) of BALB/c mice infected with *L. infantum* parasites and measured by the limiting dilution assay. Five p.o. administrations of unloaded NLC or compounds **2h** and **2m** loaded into NLC (**2h**-NLC and **2m**-NLC) at a dose of 4 mg/kg were administered every other day for 10 days. Fungizone, used as positive control, was administered during 10 consecutive days by the i.v. route at 1 mg/kg. Parasite burden was also determined at the beginning of the treatment (4 weeks after infection, Control 4w) and in untreated mice run in parallel with treated ones (6 weeks after infection, Control 6w). * *p* < 0.05, ** *p* < 0.01, *** *p* < 0.001 vs untreated mice (Control 6w), using a nonparametric Kruskal–Wallis test followed by Dunn’s multiple comparison.

The short-term efficacy of the formulations was tested in a mice model of established VL infection 4 weeks after *L. infantum* stationary-parasites inoculation. Mice received 5 p.o. administrations of compounds loaded into NLC (**2h**-NLC and **2m**-NLC) at a dose of 4 mg/kg every 2 days. Fungizone was used as positive control i.v. administered at 1 mg/kg during 10 consecutive days. As shown in Figure 7, **2m**-NLC but not **2h**-NLC p.o. reduced the parasite burden similarly to Fungizone. Healing rates of 96.9% in the liver (not significant, ns), 79.1% in the spleen, and 86.4% (*p* < 0.01) in the bone marrow for **2h**-NLC treated mice and cure rates of 99.9% (*p* < 0.001) in the liver, 98.5% (*p* < 0.001) in the spleen, and 94.8% (*p* < 0.05) in the bone marrow for **2m**-NLC treated mice were found. On the other hand, unloaded NLC (around 12 mg of lipids/mice) did not shown any effect, probably because their principal ingredients were either nonabsorbed or digested to compounds without activity after their oral administration.^31^

Finally, it has been previously described that BALB/c mice are able to control the parasite burden in the liver by the formation of granulomas, whereas they succumb to parasite infection in the spleen,^33^ although the evolution time seemed to be affected by the experimental conditions.^34^ Four or six weeks after i.v. administration of 10^8^ stationary-phase promastigotes, untreated mice showed similar parasite burden in the liver (tendency to decrease, ns), spleen and bone marrow (Control 4w vs Control 6w in Figure 7). Thus, we outlined similar efficacy for the different treatments regardless of the control mice group used for comparison (4 or 6 weeks postinfection).

### Preclinical Safety Profile

#### Compounds **2h** and **2m** Did Not Show Genotoxicity

The genotoxicity of compounds **2h** and **2m** was tested by the SOS/UMU test. Either with or without metabolic activation, compounds did not produce cytotoxicity and DNA damage at the concentrations evaluated (highest concentration of 1 mg/mL). The detailed results are summarized in the Supporting Information (Figure S2).

#### Repeated Dose Toxicity Study of Mice

**Figure 8:**
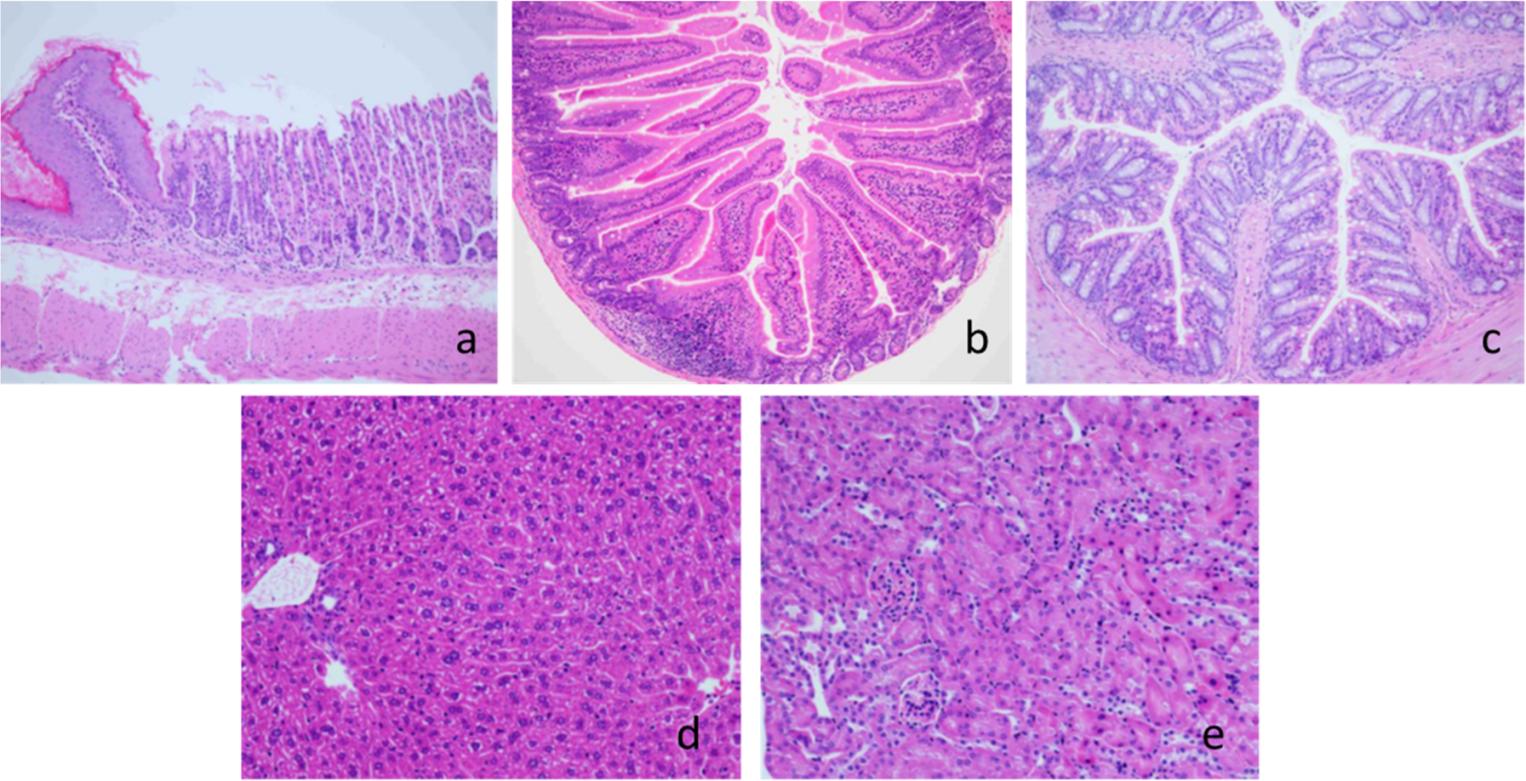
Histological samples of stomach (a), small (b) and large (c) intestine, liver (d), and kidney (e) of mice that received 5 p.o. administrations of **2m**-NLC every other day for 10 days at 4 mg/kg. Organs were fixed in 4% paraformaldehyde for 24 h, embedded in paraffin, and stained with hematoxylin-eosin. Images were taken with a 100× magnification (images a, b, and c) or a 200× magnification (images d and e).

Histopathological examination of liver, kidneys, stomach, and intestine was performed after 5 p.o. administrations every other day of **2m-**NLC at 4 mg/kg. No signs of toxicity were found in the stomach and intestines of treated mice (Figure 8a,b,c). Kidneys of mice showed mild interstitial nephritis (Figure 8e). On the contrary, liver histopathology of **2m**-NLC treated mice was normal (Figure 8d). Moreover, biochemical parameters after 5 p.o. administrations every other day at 25 mg/kg of **2m**-NLC were normal with no differences when comparing to control mice (Table 6). These findings confirmed that **2m**-NLC had a safety margin of at least 6-fold.

**Table 6:** Serum Biochemistry Evaluation for the Repeated-Dose Toxicity Study^a^

	control	**2m**-NLC
ALB^b^	3.6 ± 0.2	2.7 ± 0.4
GLU^c^	179.8 ± 15.8	237.9 ± 44.4
AST^d^	105.6 ± 14.5	116.7 ± 24.1
ALT^e^	77.5 ± 18.5	98.8 ± 67.2
CHO^f^	118 ± 2.1	128.7 ± 11.1
CREA^g^	0.17 ± 0.01	0.12 ± 0.06
TP^h^	4.8 ± 0.1	4.9 ± 0.4
BUN^i^	33.5 ± 8.5	36.7 ± 3.5

a Mice were p.o. administered with 2m-NLC every other day for 10 consecutive days at 25 mg/kg. Values are the mean ± SD, *n* = 5.

b Albumin.

c Glucose.

d Aspartate transaminase.

e Alanine aminotransferase.

f Cholesterol.

g Creatinine.

h Total protein.

i Blood urea nitrogen.

## Discussion

In spite of the great progress in proteomics, metabolism, and any omics technologies, phenotypic drug screening (PDS) still remains the most successful strategy for the identification of antileishmanial compounds,^35^ mostly due to the limited number of fully validated targets. This strategy allowed us to identify two selenocompounds (**2h** and **2m**) with IC_50_ values of 2 and 1 μM, respectively, against intramacrophage amastigotes and selectivity index higher than 20 and 50 vs THP-1 macrophages. In the current study, they were progressed for ADMET profiling. According to ADMET prediction,^19^ neither compound **2h** nor **2m** fulfilled Lipinski’s rule of 5. However, there are many approved drugs that are outside the cutoff,^36^ and this issue is especially clear for antimicrobials, probably due to their intracellular target.^37^ The *ex vivo* intestinal permeation assay (Figure 5) and *in vivo* PK study confirmed the null oral bioavailability of both selenocompounds. However, **2h** and **2m** can be categorized as stable and with an intermediate clearance according with experimentally determined *t*_1/2_ and calculated Cl_int_, respectively (Table 2). Thus, the loading of these compounds into NLC was addressed, as solubility and permeability are properties that can be very well modulated with an adequate strategy of formulation. The ability of these delivery systems for enhancing the oral bioavailability of compounds classified by the Biopharmaceutical Classification System (BCS) as class II and class IV compounds has been widely documented and specifically applied for the delivery of drugs with kinetoplastid activity.^38−41^ The almost exclusive solubility of compounds **2h** and **2m** in Transcutol,^42,43^ at least among the lipids used during the screening phase, restricted the NLC composition. This liquid lipid has been previously reported for the preparation of NLC destined to oral^44,45^ or more frequently topical administration.^46,47^ In depth, it is frequently used in topical formulations because of its excellent solubilizing and permeation enhancer properties.^48^ Furthermore, Transcutol HP, the diethylene glycol monoethyl ether (DEGEE) with the highest purity, can also be found in a number of oral prescription drugs, although the Scientific Committee on Consumer Products (SCCP) did not advise its use because recent studies indicated side effects by this administration route.^49^ However, the toxicity could be produced by impurities and only at higher exposure levels than the ones used in the current study.^50^ In fact, the amount of administered NLC corresponded to 200 mg/kg of Transcutol every other day for 10 days, whereas the no observed adverse effect level (NOAEL) was about 850–1000 mg/kg/day after a daily oral administration for 90 days. On the other hand, the NLC composition used in the current study also included a relatively high proportion (20%, Table 3) of Tween 80 that would correlate with the administration of 0.8 g/kg per dose, which is far from the NOAEL value of 10 g/kg/day, previously reported in mice after its administration for 13 weeks.^51^ Histological samples of gastrointestinal tract, kidneys (Figures 8a–c), and biochemistry of serum samples (Table 6) did not evidence any sign of toxicity after a regimen of 5 administrations of NLC every other day at a dose 6-fold higher than that administered in the efficacy study.

The PK parameters obtained after the oral administration of NLC (Table 5) confirmed the availability of these nanocarriers to allow their oral absorption, whereas no Se levels were detected after the administration of the free compounds (under the limit of detection). Therefore, the *ex vivo* intestinal permeation study (Figure 5) would indicate that, apart from the enhancement of **2h** and **2m** solubility, NLC improved their oral absorption by the increase in the permeation rates. Currently, the role of Transcutol as an oral absorption promoter has not been investigated in detail yet. However, neither the *in vivo* release nor the *ex vivo* permeability methods used in the current study were useful to accurately predict the *in vivo* behavior and PK profile of NLC, which was higher and faster. One hour after their administration, 50% of the administered dose was detected in plasma, whereas the percentage of Se released did not reach 20% at short incubation times. Actually, there is lack of robust and biorelevant *in vitro* drug release methods, especially for nanoparticle systems.^52^ Sink conditions are generally recommended to mimic the removal of released or absorbed drug by the systemic circulation; thus, a mixture of organic solvents (2% v/v PEG 400, 5% w/v d-α-tocopherol polyethylene glycol 1000 succinate and 2.5% v/v DMSO) were used for this purpose. However, permeability limitations of **2h** and **2m** compounds and their probable enterohepatic circulation complicated their PK profile and their correlation with the *in vitro* release studies.

Next, as the plasmatic Se level obtained after a single p.o. administration of **2m**-NLC formulation was higher than its leishmanicidal IC_50_ value, an *in vivo* evaluation of its efficacy was performed in a mice model of established VL after the administration of 4 mg/kg. **2h**-NLC p.o. administered were also tested for comparison. The reduction of parasite burden in the liver, spleen, and bone marrow of mice receiving **2m**-NLC was similar to that obtained after the i.v. administration of Fungizone, whereas **2h**-NLC significantly reduced the parasite load in the liver and bone marrow but not in the spleen (Figure 7).

The higher efficacy of **2m**-NLC vs **2h**-NLC would be closely associated with its higher *in vitro* intrinsic antileishmanial activity (IC_50_ of 2 μM vs 1 μM for **2h** and **2m,** respectively, Table 1) and better TR inhibition (Table 1), as their formulation into NLC presented similar effects in the permeability and oral bioavailability for both of them (Figure 5 and Figure 6). In depth, whereas Se plasmatic levels after the administration of **2h-**NLC never reached the IC_50_ levels (should be superior to 1000 μg/L), **2m**-NLC reached its IC_50_ at around 8 h (Figure 6). However, it should be pointed out that the quantification of the Se plasmatic levels by atomic absorption spectroscopy did not allow the discrimination of parent compounds and their metabolites. Moreover, plasma concentration–time curves with several peaks were obtained for all treatments (**2h** or **2m** either free or loaded into NLC). Although multiple peaking can occur as a consequence of different mechanisms, the characteristic enterohepatic circulation shown for organic Se compounds has been previously described as a way to control their body levels^53^ and the most common cause of several peaks in blood.^54^ However, a slower lymphatic absorption, especially for **2m** compound and its formulation, cannot be discarded in view of its highly lipophilic character (log*P* of 5). Furthermore, we should quantify the compounds (and/or their metabolites) in the main VL affected organs (spleen, liver, and bone marrow), as the antileishmanial efficacy of common drugs has been more closely correlated with their organ accumulation than with their plasmatic levels.^55^

Overall, the loading of the selenocompound **2m** into NLC was performed, and this formulation given orally showed similar efficacy to i.v. Fungizone. Further studies are necessary to elucidate its mechanism of hepatic clearance and perform the identification of metabolites in order to optimize the compound. Finally, we would also consider the formulation of **2m** in simpler and cheaper lipid-based delivery systems (i.e., self-emulsifying drug delivery systems) for better translation of an accessible medicine to low-resources countries.

## Conclusion

Despite its good antileishmanial activity and selectivity index, the BCS type IV compound **2m** (low solubility and low permeability) would be currently discarded for further evaluation according with the most common decision algorithm for oral drug discovery against leishmaniasis. NLC drastically modified its drug-likeness properties and rendered it bioavailable and effective after oral administration. This work is a good example of the importance of introducing delivery approaches in early drug discovery.

## Methods

### Chemicals

Compounds **2h** (*N,N′*-(4,4′-diselanediylbis(4,1-phenylene))bis-furan-2-carboxamide) and **2m** (*N,N′*-(4,4′-diselanediylbis(4,1-phenylene))bisnaphthamide) were synthesized as previously described.^19^ Briefly, compound **2h** was obtained by the reduction of *N*-(4-selenocyanatophenyl)furan-2-carboxamide with sodium borohydride in ethanol and stirring for 2 h. Compound **2m** was synthesized from bis(4-aminophenyl)diselenide and naphtoyl chloride in dry chloroform with stirring for 24 h. For their purification, **2h** was recrystallized from ethanol, whereas **2m** was only washed with diethyl ether. Both compounds were obtained with high yields (61% and 54%, respectively) and with a purity >95% confirmed by infrared spectroscopy (FTIR), nuclear magnetic resonance (^1^H NMR, ^13^C NMR), mass spectrometry, and elemental analysis.^19^ Polyethylene glycol 400 and 20,000 (PEG) were obtained by Fluka (Bucharest, Romania). Phosphate-buffered saline (PBS) was obtained by ThermoFisher Scientific (Massachusetts, USA). Glyceryl palmitoestearate (Precirol ATO5) and high purity diethylene glycol monoethyl ether (Transcutol HP) were kindly gifted by Gattefossé (Madrid, Spain). Polysorbate 80 (Tween 80) was purchased from Panreac (Illinois, USA). l-Glutamine was obtained from Acros (Geel, Belgium), and dimethyl sulfoxide (DMSO) was purchased from VWR prolabo (Llinars del Valles, Spain). d-α-Tocopherol polyethylene glycol 1000 succinate, sucrose, β-nicotinamide adenine dinucleotide phosphate reduced tretra(cyclohexylammonium) salt (NADPH), glycerol, sodium phosphate dibasic (Na_2_HPO_4_), and sodium taurocholate were purchased from Sigma (Madrid, Spain), and monosodium phosphate (NaH_2_PO_4_) from BDH Prolabo (Llinars del Valles, Spain). Lipoid S100 (soybean lecithin) was kindly gifted by Lipoid GMBH (Ludwigshafen, Germany). Acetonitrile (ACN) and water (HPLC grade) were obtained from Sigma and VWR Prolabo Chemicals, respectively. All other reagents were of analytical grade and were employed without further purification.

### Parasites

*L. infantum* infective promastigotes (strain BCN-150) were maintained at 26 °C in a continuously stirred Schneider’s modified medium (Sigma) supplemented with 20% FBS and 40 mg/mL of gentamicin (Sigma). Parasites were grown until they reached the stationary phase. Then, they were washed twice with PBS and were harvested for inoculation in BALB/c mice. Parasite infectivity was maintained by constant passages in BALB/c mice.

### Animals

Female BALB/c mice weighting between 20 and 25 g and female Wistar rats weighing 200–250 g (Harlan) were kept under normal conditions with free access to food and water. They were housed in groups of four or five in plastic cages in controlled environmental conditions (12/12 h light/dark cycle and 22 ± 2 °C). The study was conducted according to ethical standards approved by the Animal Ethics Committee of the University of Navarre in strict accordance with the European legislation in animal experiments (protocols code number 125-14E1, 127-14E1, and 100-19 approved by the Government of Navarra).

### *In Vitro* Genotoxicity Studies

*Salmonella typhimurium* (TA1535/pSK1002 strain) (DSMZ, Germany) was employed to determine the genotoxic potential of the compounds **2h** and **2m** and some of their possible metabolites, as previously described.^56^ Bacteria were exposed to different concentrations of compounds either with or without metabolic activation (S9 mix, Mutazyme) to determine the bacterial survival percentage, calculated by measuring the absorbance at 600 nm. Afterward, the β-galactosidase activity was conducted using a colorimetric method employing *o*-nitrophenyl-β-d-galactopiranoside (ONPG) as a substrate. Genotoxicity activity was measured in terms of β-galactosidase activity calculating an induction factor (IF) relative to the negative control (i.e., bacteria exposed to the solvent) and positive controls (4-nitroquinoline for normal treatments and 2-aminoantracen for S9 metabolic activated treatments). A compound was considered genotoxic for IF values ≥2 at noncytotoxic concentration (i.e., bacterial survival ≥80%). The experiment was considered valid for positive controls if IF ≥ 2.

### *In Vitro* Hepatic Microsomal Metabolism Studies

Liver microsomes were isolated as previously described with minor modifications.^57^ Mouse livers were removed and rinsed with 0.9% (w/v) NaCl solution. Then, livers were homogenized in ice-cold 0.25 M sucrose in 0.1 M PBS (pH 7.5). After 20 min of centrifugation at 9000 × *g*, the supernatant was ultracentrifuged at 100,000 × *g* for 1 h at 4 °C. The obtained microsome pellet was washed with 0.05 M PBS (pH 7.5) followed by another ultracentrifugation at 100,000 × *g* for 1 h at 4 °C. Microsomes were resuspended in 20% glycerol–0.1 M PBS and stored at −80 °C until use. Protein concentration was measured by microBCA kit (Thermo Scientific). For the metabolic assay, compounds were incubated with liver microsomes (0.5 mg protein/mL) for several time points. Stock solutions were prepared in DMSO, and the final concentration in the mixtures did not exceed 1% (v/v). The tested compounds **2h** and **2m**, at a final concentration of 500 μM, along with the microsomes were mixed in 0.05 M potassium phosphate buffer and incubated at 37 °C with continuous shaking in a final volume of 300 μL. Reaction was initiated by adding NADPH (1 mM). Controls without hepatic cofactor and blanks without compounds were also analyzed. Metabolism was determined at 0, 0.1, 0.25, 0.5, 1, 1.5, and 2 h by the addition of an equal volume of ice-cold ACN, and samples were centrifuged at 9000 × *g* for 10 min. Supernatants were stored at −20 °C until high liquid performance chromatography (HPLC) quantification analysis at 290 nm, as described in the Supporting Information. Results are expressed as mean ± SD of at least three replicates. *In vitro* half-time (*t*_1/2_, h) was established from the slope of linear regression of the percentage of remaining parent compound against time. The rate of microsomal intrinsic clearance (Cl_int,micr_, mL·min^–1^·kg^–1^) was then calculated following eq 1(27)
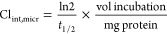
1where *t*_1/2_ was the half-life of the tested compound (h), vol was the volume of incubation (0.3 mL), and mg was the amount of protein (0.15 mg.).

The calculated Cl_int,micr_ was used for the prediction of *in vivo* intrinsic hepatic clearance (Cl_int_) using eq 2 and suitable scaling factors^27^
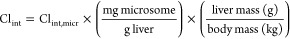
2where the scaling factors were 45 mg of microsomal protein per gram of liver tissue and 40 g of liver tissue per kilogram of body weight (mice).

### Solubility of Compounds **2h** and **2m** in Fasted Simulated Intestinal Fluid (FaSSIF)

This experiment determines the solubility of a solid compound in fasted simulated intestinal fluid (FaSSIF) at pH 6.5 after 4 h of equilibration at r.t.^58^ In brief, 1 mL of FaSSIF (3 mM sodium taurocholate and 0.75 mM lecithin in sodium phosphate buffer at pH 6.5) was added to 2 mg of the solid compounds. The resulting suspensions were shaken at 900 rpm for 4 h at r.t. Then, they were centrifuged at 12,600 × *g* for 10 min. The amount of selenocompounds, as selenium (Se) content, remaining in the supernatant was quantified by graphite atomic absorption spectrometry, as described in the Supporting Information. Results are expressed as mean ± SD of three replicates.

### Preparation of Nanostructured Lipid Carriers (NLC) Formulations

Compounds **2h** and **2m** were formulated in NLC. The preparation of NLC was made using a modification of a previously described hot high shear homogenization (HHSH) method.^12^ Briefly, 0.35 g of Precirol ATO5 was melted at a temperature 10 °C above its melting point and then mixed along with 0.15 g of Transcutol HP and 0.6 g of Tween 80. Then, 3 mg of the compounds **2h** and **2m** were added to the melted lipid mixture until complete solubilization. After that, 12 mL of purified water, previously heated at the same temperature, was added to the lipid phase and the mixture was homogenized using a high-shear laboratory mixer (Silverson SL2, UK) in the heated water bath at 12,300 rpm for 10 min to maintain the melting temperature of the lipids. The NLC dispersions were finally obtained by allowing the hot nanoemulsion to cool in an ice bath with gentle agitation for 5 min. After 24 h, samples were purified by centrifugation at 1500 × *g* for 5 min and Sephadex G-25/PD-10 columns and concentrated until 3 mL by dialysis using PEG 20,000. The final dispersions were lyophilized (Lyobeta lyophilizer, Telstar) with 10% (w/v) of trehalose and stored at 4 °C until further use.

### NLC Characterization

#### Particle Size, Polydispersity, and Surface Charge

The mean hydrodynamic diameter of the formulations and the zeta potential were determined before and after freeze-drying by photon correlation spectroscopy and electrophoretic laser Doppler anemometry, respectively, using a Zetamaster analyzer system (Malvern Instruments Ltd., Worcestershire, UK). The resuspension of freeze-dried NLC was performed in type I water with vigorous mechanical agitation. The diameter of the NLC and polydispersity index (PDI) were determined after dispersion in ultrapure water (1:100) and measured at 25 °C by dynamic light scattering angle of 90 °C. The zeta potential was determined after dilution of 200 μL of the samples in 2 mL of KCl solution (0.1 mM). Results are expressed as mean ± SD of at least three replicates.

#### Determination of Drug Loading and Entrapment Efficiency

The drug loading in both aqueous and lyophilized NLC was determined after digesting and vortexing for 10 min the NLC with ACN, which promoted the precipitation of the lipid phase. Samples were centrifuged at 4200 × *g* for 20 min. The amount of drug remained in the supernatant was then quantified by HPLC at 290 nm, as described in the Supporting Information. The drug loading (DL%) was calculated as μg of compound per mg of lipids. The entrapment efficiency (EE) was determined as percentage of drug loaded into NLC from nominal initial amount used for their preparation.

### Thermal Studies

The response to heating was studied employing a simultaneous TGA/sDTA 851e Mettler Toledo thermoanalyzer (Schwerzenbach, Switzerland). Experimental thermogravimetric and differential thermal analysis curves were obtained by monitoring about 1–5 mg of the different samples in an alumina crucible at a heating rate of 10 °C/min from 25 to 575 °C. The thermal analyses were performed under static air atmosphere using 20 mL min^–1^ of N_2_ as purge gas. Measurements were performed in triplicate.

### X-ray Diffraction Studies

X-ray diffraction studies were performed in order to study the crystalline state of compounds **2h** and **2m** and their formulations. For this purpose, samples were placed in powder form on a plastic plate in a diffractometer Bruker D8 Advance ECO (Karslruhe, Germany), with a LYNXEYE-XE-T 1D detector, using Cu Kα_1_ radiation of 1.54060 Å, a voltage of 40 kV, and a current of 30 mA, with primary and secondary soller slits. The diffraction patterns were carried out from 5° to 40° 2θ, 3 s per step, and a step size of 0.02°.

### *In Vitro* Release Studies

The release studies of compounds **2h** and **2m** were performed at 37 °C using simulated gastric fluid (SGF, pH 1.2, pepsin 0.32% (w/v)) and simulated intestinal fluid (SIF, pH 6.8, pancreatin 1% (w/v)) with 2% v/v PEG 400, 5% w/v d-α-tocopherol polyethylene glycol 1000 succinate and 2.5% v/v DMSO added to ensure sink conditions. The dialysis membranes (with 100 kDa pore size) were filled with 30 mg of lyophilized formulations either **2h**-NLC or **2m**-NLC (corresponding to around 60 μg of the selenocompounds) dispersed in 1 mL of distilled water. The loaded dialysis membranes were placed into a beaker containing 25 mL of SGF. The simulated fluid was kept under magnetic stirring at 150 rpm, and at fixed times 0.250 mL samples were withdrawn and replaced with an equal volume of SGF. Two hours after the incubation in SGF, the device was transferred to another beaker containing 25 mL of SIF magnetically stirred at 150 rpm. As previously, at fixed times, samples were withdrawn and replaced with free SIF. The *in vitro* release study finished 24 h after the experiment began. The amount of Se released from the nanocarriers was determined by graphite atomic absorption spectrometry, as described in the Supporting Information. Results are expressed as mean ± SD of at least three replicates.

### *Ex Vivo* Intestinal Permeability Studies

Intestinal permeation experiments were performed by using the Ussing chamber method as previously described.^59^ Wistar female rats were fasted for 16 h with free access to water before the experiment. Rats were anesthetized with isoflurane and sacrificed by cervical dislocation. Jejunum was aseptically extracted, excised in 2 cm segments avoiding Peyer’s patches, and placed in cold 0.9% NaCl solution. Jejunum segments were anchored into the Ussing chambers. All experiments were realized from the apical side or donor compartment (DC) to the basolateral or receptor (RC) direction. For **2h** and **2m** compounds experiments, 5 mL of deionized water at pH 7.4 containing 4% (w/v) d-α-tocopherol polyethylene glycol 1000 succinate, and 5% (w/v) PEG 400 was added to both compartments to ensure sink conditions. In the case of NLC, 5 mL of PBS was used in both compartments. Chambers were continuously oxygenated and maintained at 37 °C. After 10 min of preincubation, the solution in the DC was replaced by 5 mL of either **2h** and **2m** solutions (final concentration of 5 mM) or their lyophilized NLC diluted in PBS at a 1 mM (in selenocompound concentration). Samples of 500 μL were collected from the RC every 20 min up to 2 h and replaced by an equal volume of the RC solution. Samples were frozen at −20 °C until analysis. The membrane integrity was evaluated by the addition of yellow lucifer to the DC and the determination of its fluorescence at the end of the experiment.^60^ Experiments were carried out in triplicate for each compound and formulation. Se content in the RC was analyzed by flame atomic absorption spectrometry, as explained in the Supporting Information. The apparent permeability coefficient per unit membrane surface area (*P*_app_ (cm/s)), expressed as mean ± SD, was calculated according to eq 3
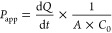
3where d*Q*/d*t* was the steady-flux (mg/s), *C*_0_ was the initial compound concentration in the DC (mg/mL), and *A* was the surface area of the membrane (cm^2^). It has been stablished that, in general, compounds with *P*_app_ > 1 × 10^–6^ cm/s are well-absorbed in the human intestinal tract whereas *P*_app_ < 1 × 10^–6^ cm/s are correlated with poor absorption.^32^

### Toxicity Studies

Acute toxicity was carried out following the Up-and-Down procedure proposed by the OECD 425.^61^ Three mice were treated with a dose of 17.5 mg/kg of each compound, **2h** and **2m**, administered by i.p. route. If all the mice died, 1/3 of the dose was administered to other new three mice. If all of them survived, the dose was tripled. If one or two of them died, the same dose was administered to one or two mice more. Both compounds were solubilized in PEG 400:H_2_O:d-α-tocopherol polyethylene glycol 1000 succinate (60:38.4:1.6, v/v/w).

For the 5-day repeated dose toxicity experiments, **2m**-NLC were p.o. administered at 4 mg/kg every other day for 5 days. Two days after the last dose, animals were sacrificed, and liver and kidneys were fixed in 4% (w/v) paraformaldehyde for 24 h, embedded in paraffin, and stained with hematoxylin-eosin (Merck) for histological observation analysis. Furthermore, body weights of **2m**-NLC treated mice were recorded prior to day 1 and prior to necropsy. Serum biochemistry was also analyzed after **2m**-NLC p.o. administered at 25 mg/kg every other day for 5 days. Blood samples were collected 2 days after the last dose. For that, blood samples were kept at r.t. for 30 min and then centrifuged at 3500 × *g* for 10 min. Serum was harvested from each blood sample, and albumin (ALB), glucose (GLU), alanine aminotransferase (ALT), aspartate aminotransferase (AST), cholesterol (CHO), creatinine (CREA), total protein (TP), and blood urea nitrogen (BUN) were measured in a Cobas biochemistry analyzer (Roche, Basel, Switzerland). Serum levels of treated mice were compared to untreated ones. Results were expressed as mean ± SD (*n* = 5).

### Pharmacokinetic Studies

A single-dose pharmacokinetic study of **2h**, **2m**, **2h**-NLC, and **2m**-NLC were evaluated in mice after i.p. (free drugs) or oral administration (free drugs and loaded into NLC). The dose (4 mg/kg) was selected taking into account LD_50_ obtained after i.p. administration of the compounds. Blood was collected from the submandibular plexus at different time points: 0, 0.25, 0.5, 1, 2, 4, 8, 24, and 48 h. Collected whole blood samples were centrifuged at 3000 × *g* for 10 min at 4 °C. The obtained plasma samples were stored at −80 °C until quantification of the drug by graphite atomic absorption spectroscopy, as described in the Supporting Information. Experiments were carried out in triplicate for each compound and formulation. Pharmacokinetic parameters were estimated by fitting the experimental data to a noncompartimental model (NCA) using PKsolver Excel program.^62^ The analyzed parameters were as follows: Area under concentration–time curve (AUC), maximum serum concentration (*C*_max_), clearance (Cl), time of maximum concentration (*t*_max_), mean residence time (MRT), and product half-life (*t*_1/2_). The extraction rate (ER) was calculated as the ratio between systemic blood clearance and mouse hepatic blood flow (considered 90 mL·min^–1^·kg^–1^). Extraction ratio can be generally classified as high (>0.7), intermediate (0.3–0.7), or low (<0.3), and these values are currently used for new chemical entities triaged during drug discovery.^63^

### Efficacy Studies in *L. infantum*-Infected BALB/c Mice

BALB/c mice were i.v. infected with 10^8^ stationary-phase promastigotes of *L. infantum* in the tail vein. Four weeks after infection, mice were divided into the following groups: (i) negative control (PBS, p.o.), (ii) unloaded NLC (p.o.), (iii) **2h**-NLC (4 mg/kg of 2h, p.o.), (iv) **2m**-NLC (4 mg/kg of 2m, p.o.), and (v) positive control (1 mg/kg Fungizone, i.v.). Fungizone was daily administered for 10 consecutive days, whereas oral treatments were given every other day for 10 days (5 treatments).

Levels of parasite burden in liver, spleen, and bone marrow were determined at the beginning and the end of the treatments by limiting the dilution assay (LDA), as previously described.^64^ Briefly, the liver, spleen, and bone marrow samples perfused from the femur cavities of each mouse were individually homogenized in Schneider medium supplemented with 10% heat-inactivated FBS and antibiotics (100 U/mL of penicillin, 100 μg/mL of streptomycin) and filtered through 40 μm cell strainers (Corning Gmbh, Germany) to obtain a cell suspension. Cells were serially diluted (1/3) in 96-well flat-bottomed microtiter plates (Thermo Fischer Scientific) containing the same medium employed for homogenization (6 replicates). The number of viable parasites was calculated as the geometric mean of the titer obtained from 6 replicates cultures × reciprocal fraction of the homogenized organ added to the first well. The titer was the reciprocal value of the highest dilution at which promastigotes were observed with an inverted light microscope after 7 days of incubation at 26 °C. Results are indicated per mg of spleen and liver, or parasites per 1 × 10^6^ BM cells.^65^

### Statistical Analysis

Statistical analyses between multiple groups were performed by using Kruskal–Wallis (nonparametric) followed by Dunn’s multiple comparisons tests. Differences between the two groups were analyzed by an unpaired *t* test. GraphPad Prism7 version (GraphPad Software, Inc., San Diego, CA, USA) was used to perform the analyses. Significance was established for a *p* value <0.05.

## Supporting Information Available

Detailed methodology of the lyophilization process and quantification of the two selenocompounds as well as results regarding the microsomal metabolism and genotoxicity (PDF)
