# Supplementary material for: Oral Efficacy of a Diselenide Compound Loaded in Nanostructured Lipid Carriers in a Murine Model of Visceral Leishmaniasis
Source: ACS Infect Dis. 2021 Nov 12;7(12):3197–209. doi: 10.1021/acsinfecdis.1c00394 (PMC8675869; doi:10.1021/acsinfecdis.1c00394)
Supplement: Supplementary file 1 — id1c00394_si_001.pdf [file id1c00394_si_001.pdf]

**Supporting Information for**

# Oral efficacy of a diselenide compound loaded in nanostructured lipid carriers in a murine model of visceral leishmaniasis

Mikel Etxebeste-Mitxeltorena[#], Esther Moreno[#], Manuela Carvalheiro, Alba Calvo, Iñigo Navarro-Blasco, Elena González-Peñas, José I. Álvarez-Galindo, Daniel Plano, Juan M. Irache, Antonio J. Almeida, Carmen Sanmartín, Socorro Espuelas*

* Corresponding author:
Institute of Tropical Health, Department of Pharmaceutical Technology and Chemistry, School of Pharmacy and Nutrition, University of Navarra, Irunlarrea 1, 31008 Pamplona, Spain. Instituto de Investigación Sanitaria de Navarra (IdiSNA), Irunlarrea 3, 31008 Pamplona, Spain. E-mail: sespuelas@unav.es

## Table of contents

Figure S1. Microsomal metabolism of 2h and 2m compounds....S2
Figure S2. Induction factor (IF) in the SOS/UMU test....S3
Lyophilization of NLC....S4
Quantification of selenocompounds ....S4-S5

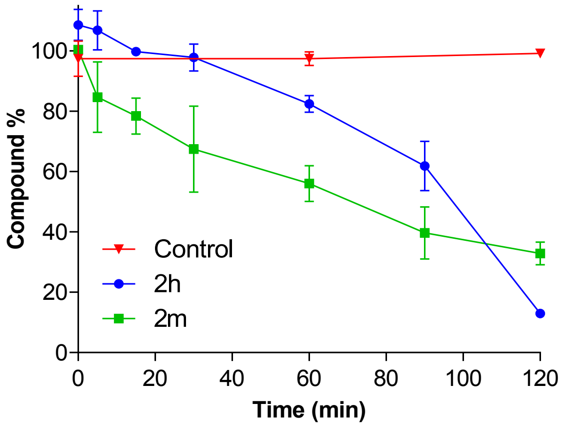


**Figure S1. Microsomal metabolism of compounds 2h and 2m at different times.** Compounds **2h** and **2m** at 500 µM were incubated at 37 ºC for different time points with BALB/c mice liver microsomes (0.5 mg protein/mL) and NADPH as cofactor. The reaction was finished by the addition of ice-cold acetonitrile and, after centrifugation, supernatants were collected and subjected to HPLC quantification. Mixtures without NADPH were employed as control. Experiments were performed in triplicate.

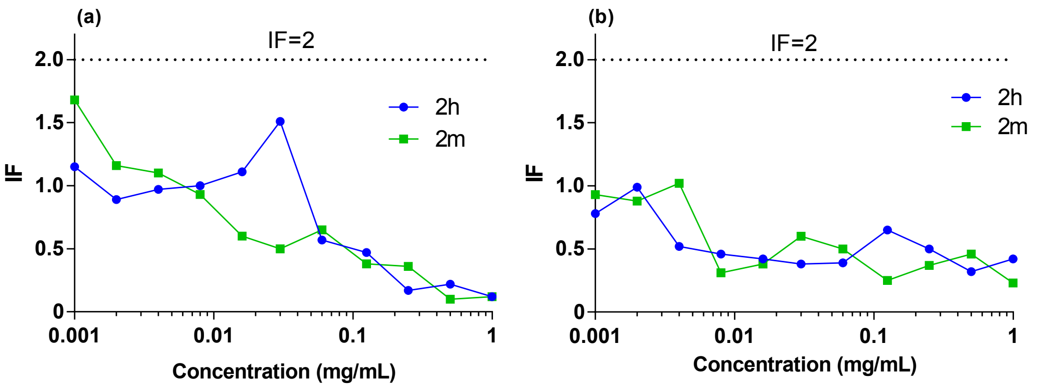


**Figure S2. Induction factor (IF) in the SOS/UMU test.** IF at different concentrations of compounds **2h** and **2m** without metabolic activation (a) or with metabolic activation (b).

***Lyophilization of nanoestructured lipid carriers (NLC)***

3 mL of NLC were added to 10 mL vials containing 10% (w/v) of trehalose and the following lyophilization protocol was used. Specifically, the formulations were slowly frozen at a shelf temperature of – 40 °C for 2 h. Primary drying was performed at a shelf temperature of – 30 °C and a pressure of 0.145 mbar for 12 h, Then, the shelf temperature was gradually increased to 5 °C during 12 h. After this period, the pressure was reduced to 0 mbar for 15 h. Finally, secondary drying was conducted at 15 °C for 33 h without vacuum. NLC were reconstituted with type I water using a vortex, and the particle size and PDI were determined.

***Quantification of selenocompounds***

The amount of the selenocompounds **2h** and **2m** in the NLC formulations and in the hepatic microsomal metabolic studies was quantified by HPLC (Hitachi System LaCrom Elite, Beckman Instruments, Inc.) at 290 nm. The analytical column was a Phenomenex® C18 (5 µm) 110A 130 x 4.5. The mobile phase consisted of a mixture of water-acetonitrile (60:40 (v/v) for compound **2h** and 70:30 (v/v) for compound **2m**) with a flow rate of 1 mL/min at 40 °C. The linearity was established between 50 and 0.5 µg/mL with a $R^2$ of 0.999. Limits of quantification and detection were 3.292 µg/mL and 1.086 µg/mL, respectively, with a 95 % of confidence.

For the solubility studies in fasted simulated intestinal fluid (FaSSIF), the *in vitro* release studies, the *ex vivo* intestinal permeation studies and the pharmacokinetic (PK) studies, the quantification of both selenocompounds were based on their selenium (Se) content and their quantification by atomic absorption spectrometry. All measurements, except for permeation studies, were carried out using a Perkin Elmer AAnalyst 800 (Norwalk, CT, USA) equipped with a longitudinal Zeeman-effect background corrector, a transversely heated Graphite Furnace Atomizer with pyrolytically coated tubes with integrated platform and an AS-800 autosampler. A Se electrodeless discharge lamp was used as the radiation source and operated with a current of 290 mA (wavelength 196.0 nm, spectral band width 0.7 nm). A solution containing palladium and magnesium nitrate was used as a modifier for Se determination. The heating program of the graphite tube was optimized to determine the Se in samples of study. A total of 30 µL (20 µL sample and 10 µL matrix modifier) was injected into the graphite tube in triplicate. All measurements were made with at least three replicates and are based on integrated absorbance. High-purity argon (99.999 %) was used as the purge gas throughout at a flow rate of 250 mL/min (stop-flow during atomization). Samples were appropriately diluted by mixing with 1% nitric acid solution. In order to verify the accuracy of the analytical methodology, a recovery study (n=6) was performed to found between 94 and 103 %. In addition, the detection limit (LOD) was calculated according to the criteria established by IUPAC ($X_b \pm 3$ s.d.$_b$) as the average of three times the standard deviation of the reagent blank, setting at 1.5 µg $L^{-1}$ equivalent to 15 µg $L^{-1}$ of

Se when expressed in terms of analyzed samples. Both blank reagent and a quality control (12.3 ± 0.9 μg $L^{-1}$, range = 11.3 – 13.1, n = 12; quality control concentration 12.4 μg $L^{-1}$) were run before analyzing the samples to check the reliability of measurements.

Regarding the intestinal permeation studies, compound concentration in the receptor compartment (RC) was measured by flame atomic absorption spectrophotometry. Appropriate dilution (1:10) of samples with 1 % nitric acid solution in acid-washed polyethylene tubes was carried out before analyzing. Measurements were accomplished by direct calibration using acidified working aqueous standards (0-120 mg $L^{-1}$) made up each day by dilution from stock standard solution (1000 mg $L^{-1}$, Merck, Darmstadt, Germany) in 1% nitric acid solution. Blank reagents were subjected to similar sample acid-dilution procedure. Detection limit (LOD) was setting at 0.51 mg $L^{-1}$ equivalent to 2.6 mg $L^{-1}$. The accuracy was checked by performing a Se recovery (n = 6) at different levels of concentration. The percentage of recoveries was satisfactory, ranging from 96 % to 102 %. Moreover, throughout the course of the study, both blank reagent and an in-house control (59.6 ± 0.4 mg $L^{-1}$, range = 58.6 – 60.7, n = 12) were run to satisfy the criteria established in the quality program and to provide on-going quality control information.
